# Antimicrobial stewardship in the era of the COVID-19 pandemic: A systematic review protocol on the opportunities and challenges for Sub-Saharan Africa

**DOI:** 10.1097/MD.0000000000033697

**Published:** 2023-05-12

**Authors:** John Njuma Libwea, Che Henry Ngwa, Armelle Viviane Ngomba, Frankline Sevidzem Wirsiy, Limkile Mpofu, Chanceline Bilounga Ndongo, Sinata Koulla-Shiro, Stephen Graham, Lionelle Patricia Tchokokam Djieuya, Nadia Mandeng, Georges Alain Etoundi Mballa, Eman Sobh, Bright I. Nwaru, Paul Koki Ndombo, Emilienne Epee

**Affiliations:** a Directorate for Disease Control, Epidemics and Pandemics Ministry of Public Health, Yaoundé, Cameroon; b Health Sciences Unit, Faculty of Social Sciences, Tampere University, Tampere, Finland; c African Science Frontiers Initiatives [ASFI], Lagos, Nigeria; d Public Health Emergency Operation Center, Yaoundé, Cameroon; e Department of Epidemiology and Population Health, Faculty of Health Sciences, American University of Beirut, Beirut, Lebanon; f Africa Centres for Disease Control and Prevention, Addis Ababa, Ethiopia; g Emerging Threats Epidemiology Group (ETEG); Department of Epidemiology| College of Public Health, University of Nebraska Medical Center (UNMC), 984395 Nebraska Medical Center, Omaha, Nebraska, USA; h Health Economics, HIV/AIDS, Research Division (HEARD), University of Kwazulu Natal, Durban, South Africa; i Faculty of Medicine and Biomedical Sciences, University of Yaoundé I, Yaounde, Cameroon; j Centre Pasteur du Cameroun, Yaounde, Cameroon; k Centre for International Child Health, University of Melbourne Department of Paediatrics and Murdoch Children’s Research Institute, Royal Children’s Hospital, Melbourne, Australia; l Chest Diseases Department, Faculty of Medicine for Girls, Al-Azhar University, Cairo, Egypt; m Respiratory Therapy Department, College of Medical Rehabilitation Sciences, Taibah University, Medina, Saudi Arabia; n Krefting Research Centre, Institute of Medicine, University of Gothenburg, Gothenburg, Sweden; o Mother and child Hospital, Chantal Biya Foundation, Yaoundé, Cameroon.

**Keywords:** antimicrobial resistance, antimicrobial stewardship, COVID-19, Sub-Saharan Africa, systematic review

## Abstract

Antimicrobial resistance (AMR) remains one of the leading threats to global public health and this may increase following COVID-19 pandemic. This is particularly the case in Africa where regulations on antimicrobial usage are weak. This protocol outlines the steps to undertake a systematic review to synthesize evidence on drivers of AMR and evaluate existing approaches to strengthening antimicrobial stewardship (AMS) programs in Sub-Saharan Africa (SSA). On the basis of the evidence generated from the evidence synthesis, the overarching goal of this work is to provide recommendations to support best practices in AMS implementation in SSA.

**Methods:**

A systematic search will be conducted using the following databases: Global Health Library, PubMed, Cumulative Index to Nursing and Allied Health Literature, Scopus, Google Scholar, Global Health, Embase, African Journals Online Library, Web of Science, antimicrobial databases (WHO COVID-19, TrACSS, NDARO, and JPIAMR), and the Cochrane databases for systematic reviews. Studies will be included if they assess AMR and AMS in SSA from January 2000 to January 31, 2023.

**Results:**

The primary outcomes will include the drivers of AMR and approaches to AMS implementation in SSA. The Preferred Reporting Items for Systematic Reviews and Meta-analyses will guide the reporting of this systematic review.

**Conclusions:**

The findings are expected to provide evidence on best practices and resource sharing for policy consideration to healthcare providers and other stakeholders both at the local and international levels. Additionally, the study seeks to establish drivers specific to AMR during the COVID-19 era in the SSA, for example, with the observed increasing trend of antimicrobial misuse during the first or second year of the pandemic may provide valuable insights for policy recommendation in preparedness and response measures to future pandemics.

**PROSPERO registration number:**

CRD42022368853.

## 1. Introduction

Antimicrobial resistance (AMR) is one of the greatest threats to global public health in the coming decades.^[1]^ AMR is “a secret global disaster” and the perfect example of the complex, multi-sectoral, multi-stakeholder dilemma we will progressively face in the future.^[1]^ Addressing drug resistance challenges demands not just scientific research but also overcoming many complex ethical issues.^[2]^ Currently, AMR is a global public health problem, especially in low- and middle-income countries, including those of Sub-Saharan Africa (SSA), where there is limited control of antimicrobial use.^[1,3]^ The failure of most communities with a disproportionately high illness burden to react to treatment with fourth generation antimicrobials, particularly among disadvantaged populations, is indicative of the immediate repercussions.^[4]^

The role of human and animal health professionals in regulating antimicrobials use cannot be overemphasized.^[5]^ For example, an average of 35% of all antimicrobial prescriptions in emergency care hospitals were deemed inappropriate or not needed.^[3]^ In view of this, a tripartite agreement was established in 2015 between the World Health Organization (WHO), the Organization for Animal Health, and the Food and Agriculture Organization (FOA), as well as with the United Nations Environment Program, to jointly combat AMR.^[6]^ This agreement was the brainchild of the “One Health Approach,” which is a unique platform involving human, animal and environmental health professionals to promote antibiotic stewardship (AMS) campaigns. The impact of antimicrobial consumption in the agricultural sector on human health has been demonstrated within the human food chain as well as in the environment.^[3,6]^ This has consequently led to poor clinical outcomes, rising healthcare expenses, and, most critically, the emergence and reemergence of multidrug-resistant pathogens that constitute a serious threat to public health.^[7,8]^ This concord with the much acclaimed O’Neill report of 2015, which had earlier emphasized that “if nothing is done by 2050, AMR will be responsible for 4.5 million deaths in LMIC, especially in SSA.”^[9]^

The term “AMS programme” refers to multidisciplinary interventions that include patient-level stewardship (e.g., optimizing antimicrobial therapy for an individual patient based on culture results and clinical syndrome) and population-level stewardship (e.g., interventions) can reduce overall antimicrobial consumption or consumption of a specific antimicrobial class.^[1,8,10]^ The merging of the patient-level- and the population-level components of AMS is believed to guarantee an optimal level of antimicrobial use in acute care settings and beyond.^[3]^

In Western countries, efforts towards regional AMS implementation have successfully led to the development of practical guidelines for AMS as well as sustaining country-specific action plans on AMR.^[11,12]^ Additionally, collaborative efforts such as the Transatlantic Taskforce on Antimicrobial Resistance sponsored by the United States Centers for Disease Control and Prevention and the European Center for Disease Prevention and Control have strengthened the need to develop more detailed AMS programs.^[13]^

However, in the SSA region, multi-sectoral collaboration at the country level to address AMR has gained ground in addition to the collaborative efforts of Africa CDC over the past decade. How this collaboration transcends into enhancing a SSA regional AMS program component remains unclear. To achieve a clearer and comprehensive understanding of the underlying evidence driving AMR and AMS in SSA, there is need to appraise the underlying evidence published in the extant literature. Furthermore, since December 2019 following the advent of the coronavirus disease-19 (COVID-19) pandemic, several new studies have been published on the topic, including those associated with COVID-19 surging antimicrobial misuse.^[1,14,15]^ In addition, self-medication with antimicrobials, which contributes to accelerating the emergence and spread of AMR is very common in Africa and varies amongst sub-regions, with Western Africa reporting the highest prevalence.^[16]^ Hence, there is need to integrate these studies to provide a contemporary synthesis of the evidence to fill gaps in the global health security agenda portfolio on AMR. Therefore, we aim to conduct a systematic review of available literature on AMR drivers and understand the factors influencing the establishment of a regional framework for AMS implementation in the SSA.

## 2. Rationale

AMR remains an endemic challenge in most low- and middle-income countries. Effective AMS programs have been available for many decades. Still, its implementation has been hampered by many factors, especially within SSA. With this review, we expect to support existing knowledge about the global health security state on AMR currently available. Furthermore, we intend to provide evidence-based recommendations that support best practices for a SSA regional AMS program implementation.

### 2.1. Review question

What are the drivers of AMR and approaches to strengthen AMS program implementation in SSA in the COVID-19 era?

## 3. Aims and objectives

In an effort to redress gaps in previous systematic reviews by incorporating new individual studies and systematic reviews in the COVID-19 era, we aim to comprehensively identify, critically appraise, and synthesize evidence from studies investigating the drivers of AMR and synthesize evidence needed to transition from national AMS programs to the implementation of a SSA regional AMS. The results of this review will contribute to existing knowledge of the problem, guide policymakers to strengthen their national AMS programs in the SSA, and provide useful information for further research in this field.

### 3.1. Specific objectives

The specific objectives are to synthesize evidence on:

1. To determine the drivers of AMR in the SSA region;
2. To determine drivers specific to AMR during the COVID-19 era in the SSA region;
3. To identify evidence-based recommendations that support sustainable engagement and resource sharing on best practices for a regional AMS program implementation in SSA.

## 4. Methods

A systematic review will be conducted and reported according to the Preferred Reporting Items for Systematic Reviews (PRISMA) guidelines.^[17]^

### 4.1. Criteria for inclusion and exclusion

We will search for randomized control trials and observational studies (including cohort, cross-sectional, and case-control), systematic reviews (for reviewing and extraction of individual studies) and conference abstracts that assessed the impact of antimicrobial prescribing, misuse, patterns of antimicrobial misuse and/or outline steps towards antimicrobial stewardship (AMS). Case reports, discussion papers or gray literature and editorials will not be included in our literature search. However, there will be no language restrictions as we will search for all relevant literature or studies published from January 1st, 2000 to January 31st, 2023 and that were conducted in or reported on data from the SSA region.

### 4.2. Study outcomes

The primary outcomes for this systematic review analysis will be the drivers of AMR and AMS implementation outcomes. AMR outcomes include antimicrobial misuse, exposure, and consumption.

### 4.3. Source of information

We will search the Global Health Library, Embase, PubMed, Cumulative Index to Nursing and Allied Health Literature, Scopus, Google Scholar, Global Health, African Journals Online Library, Web of Science, antimicrobial databases including (WHO COVID-19, Tripartite AMR Country Self-Assessment Survey, National Database of Antibiotic Resistant Organisms and The Joint Programming Initiative on Antimicrobial Resistance) and the Cochrane databases for systematic reviews. Moreover, the search for gray literature will be performed through sources such as ProQuest Dissertations and Theses Global, Mascot/Wotro, Effective Public Health Practice Projects, Public Health Gray Literature Sources and Health Evidence.

### 4.4. Search strategies

We will conduct an electronic systematic article search from the above electronic databases for relevant studies based on the research question “What are the drivers of AMR and approaches to strengthen AMS program implementation in SSA in the COVID-19 era?” The following concepts will be used:

Concept 1: AMS programs

□ “Antimicrobial stewardship” OR “Antimicrobial policies” OR “Antimicrobial intervention program*” OR “Antimicrobial surveillance systems” OR “Antimicrobial action plan*” OR “antimicrobial stewardship”[MeSH Terms]

Concept 2: Drivers or risk factors of AMR

□ “Antimicrobial resistance” OR “antimicrobial resistan*” OR amr OR “Microbial drug resistant” OR “Multidrug resistant” OR mdr OR “Multiple drug resistant” OR “Antibiotic resistant” OR abr OR “Antibiotics resistant” OR “Antibacterial resistant” OR “Bacteria drug resistan*” OR “Antimicrobial susceptibility” OR Drivers OR “Risk factors” OR “anti-infective agents” OR “anti-infective agents”[MeSH Terms]

Concept 3: COVID-19

□ “COVID-19” OR “COVID-19”[MeSH Terms] OR “COVID-19 Vaccines” OR “COVID-19 Vaccines”[MeSH Terms] OR “COVID-19 serotherapy” OR “COVID-19 Nucleic Acid Testing” OR “covid-19 nucleic acid testing”[MeSH Terms] OR “COVID-19 Serological Testing” OR “covid-19 serological testing”[MeSH Terms] OR “COVID-19 Testing” OR “covid-19 testing”[MeSH Terms] OR “SARS-CoV-2” OR “Severe Acute Respiratory Syndrome Coronavirus 2” OR “NCOV” OR “2019 NCOV” OR “2019-nCoV” OR “Wuhan coronavirus” OR “SARS-CoV-2”[MeSH Terms]

Concept 4: SSA countries

□ Africa OR “Africa south of the Sahara”[MeSH Terms] OR “Sub-Saharan Africa” AND (“Countries” OR “Country” OR “country” OR “countries”) OR (Cameroon OR “Central African Republic” OR Chad OR Congo OR “Democratic Republic of the Congo” OR “Equatorial Guinea” OR Gabon OR “Sao Tome and Principe” OR Burundi OR Djibouti OR Eritrea OR Ethiopia OR Kenya OR Rwanda OR Somalia OR “South Sudan” OR Sudan OR Tanzania OR Uganda OR Angola OR Botswana OR Eswatini OR Lesotho OR Malawi OR Mozambique OR Namibia OR “South Africa” OR Zambia OR Zimbabwe OR Benin OR “Burkina Faso” OR “Cabo Verde” OR “Cote d’Ivoire” OR Gambia OR Ghana OR Guinea OR “Guinea-Bissau” OR Liberia OR Mali OR Mauritania OR Niger OR Nigeria OR Senegal OR “Sierra Leone” OR Togo)

### 4.5. Screening of retrieved literature

The literature retrieved from the databases will be transferred to Endnote for the removal of duplicate papers. Thereafter, the papers will be exported to Rayyan for further screening, and each title and/or abstract will be screened independently by at least 2 coauthors for potentially eligible studies. The full texts of selected papers will be obtained for screening, if discrepancies arise between the 2 coauthors, it will be resolved through discussion between them. If no consensus is reached, a third coauthor will arbitrate. The PRISMA flow chart will be used to report the screening process.

### 4.6. Registration and reporting

This study protocol has been registered in October 2022 with the University of York Center for Reviews and Dissemination of the International Prospective Register of Systematic Reviews (registration number is CRD42022368853). The protocol is reported according to the PRISMA-P guidelines for reporting of systematic review protocols.

### 4.7. Data extraction

A standardized data extraction form will be developed. It will be used by at least 2 of the coauthors to independently extract relevant study data from eligible studies. Before full use with all included studies, the data extraction form will first be piloted using a couple of the selected studies. Following the piloting, necessary amendments will be made to the extraction form and will be used for data extraction of all the relevant studies. Information collected from each study will include the title, year of publication, authors, country, study design, description of the intervention, and the description of AMS outcomes.

### 4.8. Quality assessment

Quality assessment of included studies will be performed independently by at least 2 coauthors using the Cochrane Risk of Bias tool for randomized controlled trials and the Newcastle-Ottawa Quality Assessment Scale for nonrandomized studies.^[18,19]^ Disagreement between the coauthors will be resolved by discussion, with the involvement of a third coauthor for a final opinion, if needed.

### 4.9. Expected outcomes and prioritization

We will report the total number of studies identified through the bibliographic database searches, as well as screening to consider those that will be relevant. After this, we will exclude studies that do not meet the inclusion criteria. The full text of all eligible studies will further be reviewed to identify those to be included in our analyses. The primary outcomes of this systematic review are to evaluate/determine the drivers of AMR and AMS implementation in the SSA region. The secondary outcome will be to synthesize evidence on best practices as well as on needed policies to enhance the establishment of a SSA regional AMS framework.

### 4.10. Data synthesis

Studies will be grouped based on both outcomes (i.e., AMR determinants and challenges of implementing a regional AMS in SSA). Our primary analysis will consider all studies conducted on AMR and AMS at the start of January 2000 until end of December 2022. Based on the characteristics of the included studies, considerations on whether to stratify our results on one or more criteria will be given, for example, AMR or AMS published before or in the era of COVID-19. We will use the following approaches: tabulation and thematic analysis. The analysis focuses on thematically grouping the barriers identified in the included studies. We will further formulate tables to capture descriptive information and data for each study to be included.

### 4.11. Discussion

In this systematic review protocol on the drivers of AMR and AMS implementation in SSA, the different types of studies, population, interventions and outcomes have been succinctly described in accordance to the research question as well as the data sources, search strategy, data extraction, methodological quality of the studies, data synthesis approach, risk of bias assessment and reporting.^[17]^ Moreover, using the “Population/Intervention (Exposure)/Comparison (Comparator)/Outcome (PICO/PEO)” concept,^[20,21]^ the research question for this review will be conceptualized to ensure a robust and systematic search of the available relevant literature.

The findings are expected to provide evidence on best practices and resource sharing for policy consideration to healthcare providers and other stakeholders both at the local and international levels. Additionally, the study seeks to establish drivers specific to AMR during the COVID-19 era in the SSA e.g., with the observed increasing trend of antimicrobial misuse during the first or second year of the pandemic may provide valuable insights for policy recommendation in preparedness and response measures to future pandemics.

More so, AMR has been described as the invisible pandemic whose effects have been amplified following the landfall of the COVID-19 pandemic, especially in SSA with weakened health systems, unregulated drug prescription channels, self-medication and usage of unlicensed herbal “therapies.” Thus, considering that local herbal therapies or traditional forms of medications may not be widely documented, their contribution to the AMR burden and subsequently, AMS enforcement might be one of the main caveats of this systematic review and this may jeopardize the external validity of the study. However, in using the PRISMA guidelines,^[17]^ we remain confident this will assure the transparency of the study at every stage.

### 4.12. Conclusion

AMR remains a global public health threat and it requires a collective effort to improve antimicrobial use across the healthcare continuum and beyond.^[22]^ Therefore, understanding the drivers and how sub-regional regulatory bodies could be established as well as implemented will go a long way to enhance the strides made by Africa CDC and the WHO.

### 4.13. Ethical considerations and dissemination

Ethical approval will not be required since we will be using secondary data (and no patients or members of the public will be involved) for this systematic review study. We intend to submit the results of this review to peer-reviewed journals on AMR and AMS, as well as present our findings at national, regional, and international scientific meetings, conferences, and/or seminars in the subject areas.

## Author contributions

**Conceptualization:** John Njuma Libwea, Stephen Graham, Georges Alain Etoundi Mballa, Bright I. Nwaru, Epee Emilienne.

**Data curation:** Lionelle Patricia Tchokokam Djieuya.

**Funding acquisition:** John Njuma Libwea.

**Investigation:** John Njuma Libwea.

**Methodology:** John Njuma Libwea, Che Henry Ngwa, Frankline Sevidzem Wirsiy, Limkile Mpofu, Stephen Graham, Eman Sobh, Bright I. Nwaru.

**Project administration:** John Njuma Libwea, Vivienne Ngomba Armelle, Frankline Sevidzem Wirsiy, Sinata Koulla-Shiro, Stephen Graham, Nadia Mandeng, Georges Alain Etoundi Mballa, Eman Sobh, Bright I. Nwaru, Paul Koki Ndombo, Epee Emilienne.

**Resources:** John Njuma Libwea, Che Henry Ngwa, Vivienne Ngomba Armelle, Frankline Sevidzem Wirsiy, Chanceline Bilounga Ndongo, Sinata Koulla-Shiro, Stephen Graham, Lionelle Patricia Tchokokam Djieuya, Nadia Mandeng, Georges Alain Etoundi Mballa, Eman Sobh, Bright I. Nwaru, Paul Koki Ndombo, Epee Emilienne.

**Software:** John Njuma Libwea, Che Henry Ngwa, Vivienne Ngomba Armelle, Frankline Sevidzem Wirsiy, Limkile Mpofu, Chanceline Bilounga Ndongo, Sinata Koulla-Shiro, Lionelle Patricia Tchokokam Djieuya, Nadia Mandeng, Georges Alain Etoundi Mballa, Eman Sobh, Bright I. Nwaru, Paul Koki Ndombo, Epee Emilienne.

**Supervision:** John Njuma Libwea, Che Henry Ngwa, Vivienne Ngomba Armelle, Frankline Sevidzem Wirsiy, Limkile Mpofu, Chanceline Bilounga Ndongo, Sinata Koulla-Shiro, Stephen Graham, Nadia Mandeng, Georges Alain Etoundi Mballa, Eman Sobh, Bright I. Nwaru, Paul Koki Ndombo, Epee Emilienne.

**Validation:** John Njuma Libwea, Che Henry Ngwa, Vivienne Ngomba Armelle, Frankline Sevidzem Wirsiy, Limkile Mpofu, Chanceline Bilounga Ndongo, Sinata Koulla-Shiro, Stephen Graham, Lionelle Patricia Tchokokam Djieuya, Nadia Mandeng, Georges Alain Etoundi Mballa, Eman Sobh, Bright I. Nwaru, Paul Koki Ndombo, Epee Emilienne.

**Visualization:** John Njuma Libwea, Che Henry Ngwa, Vivienne Ngomba Armelle, Frankline Sevidzem Wirsiy, Limkile Mpofu, Chanceline Bilounga Ndongo, Sinata Koulla-Shiro, Stephen Graham, Lionelle Patricia Tchokokam Djieuya, Nadia Mandeng, Georges Alain Etoundi Mballa, Eman Sobh, Bright I. Nwaru, Paul Koki Ndombo, Epee Emilienne.

**Writing – original draft:** John Njuma Libwea, Che Henry Ngwa, Stephen Graham.

**Writing – review & editing:** John Njuma Libwea, Che Henry Ngwa, Vivienne Ngomba Armelle, Frankline Sevidzem Wirsiy, Chanceline Bilounga Ndongo, Sinata Koulla-Shiro, Stephen Graham, Lionelle Patricia Tchokokam Djieuya, Nadia Mandeng, Georges Alain Etoundi Mballa, Eman Sobh, Bright I. Nwaru, Paul Koki Ndombo, Epee Emilienne.
